# Therapeutic Potential of Isoflavones with an Emphasis on Daidzein

**DOI:** 10.1155/2021/6331630

**Published:** 2021-09-09

**Authors:** Mohammed M. Alshehri, Javad Sharifi-Rad, Jesús Herrera-Bravo, Evelyn L. Jara, Luis A. Salazar, Dorota Kregiel, Yadav Uprety, Muhammad Akram, Mehwish Iqbal, Miquel Martorell, Margalida Torrens-Mas, Daniel Gabriel Pons, Sevgi Durna Daştan, Natália Cruz-Martins, Fethi Ahmet Ozdemir, Manoj Kumar, William C. Cho

**Affiliations:** ^1^Pharmaceutical Care Department, Ministry of National Guard-Health Affairs, Riyadh, Saudi Arabia; ^2^Phytochemistry Research Center, Shahid Beheshti University of Medical Sciences, Tehran, Iran; ^3^Departamento de Ciencias Básicas, Facultad de Ciencias, Universidad Santo Tomas, Chile; ^4^Center of Molecular Biology and Pharmacogenetics, Scientific and Technological Bioresource Nucleus, Universidad de La Frontera, Temuco 4811230, Chile; ^5^Department of Environmental Biotechnology, Lodz University of Technology, Wolczanska 171/173, 90-924 Lodz, Poland; ^6^Amrit Campus, Tribhuvan University, Kathmandu, Nepal; ^7^Department of Eastern Medicine and Surgery, Directorate of Medical Sciences, GC University Faisalabad, Pakistan; ^8^Institute of Health Management, Dow University of Health Sciences, Karachi, Pakistan; ^9^Department of Nutrition and Dietetics, Faculty of Pharmacy and Centre for Healthy Living, University of Concepción, 4070386 Concepción, Chile; ^10^Translational Research In Aging and Longevity (TRIAL Group), Health Research Institute of the Balearic Islands (IdISBA), 07122 Palma, Spain; ^11^Grupo Multidisciplinar de Oncología Traslacional (GMOT), Institut Universitari d'Investigació en Ciències de la Salut (IUNICS), Universitat de les Illes Balears (UIB), Instituto de Investigación Sanitaria Illes Balears (IdISBa), 07122 Palma, Spain; ^12^Department of Biology, Faculty of Science, Sivas Cumhuriyet University, 58140 Sivas, Turkey; ^13^Beekeeping Development Application and Research Center, Sivas Cumhuriyet University, 58140 Sivas, Turkey; ^14^Faculty of Medicine, University of Porto, Alameda Professor Hernâni Monteiro, 4200-319 Porto, Portugal; ^15^Institute for Research and Innovation in Health (i3S), University of Porto, 4200-135 Porto, Portugal; ^16^Institute of Research and Advanced Training in Health Sciences and Technologies (CESPU), Rua Central de Gandra, 1317, 4585-116 Gandra, PRD, Portugal; ^17^Department of Molecular Biology and Genetics, Faculty of Science and Art, Bingol University, Bingol 1200, Turkey; ^18^Chemical and Biochemical Processing Division, ICAR–Central Institute for Research on Cotton Technology, Mumbai 400019, India; ^19^Department of Clinical Oncology, Queen Elizabeth Hospital, Kowloon, Hong Kong

## Abstract

Daidzein is a phytoestrogen isoflavone found in soybeans and other legumes. The chemical composition of daidzein is analogous to mammalian estrogens, and it could be useful with a dual-directional purpose by substituting/hindering with estrogen and estrogen receptor (ER) complex. Hence, daidzein puts forth shielding effects against a great number of diseases, especially those associated with the control of estrogen, such as breast cancer, diabetes, osteoporosis, and cardiovascular disease. However, daidzein also has other ER-independent biological activities, such as oxidative damage reduction acting as an antioxidant, immune regulator as an anti-inflammatory agent, and apoptosis regulation, directly linked to its potential anticancer effects. In this sense, the present review is aimed at providing a deepen analysis of daidzein pharmacodynamics and its implications in human health, from its best-known effects alleviating postmenopausal symptoms to its potential anticancer and antiaging properties.

## 1. Introduction

Nutraceuticals contain selective combinations of specific plant-derived bioactive components with renowned medicinal, disease-preventing, and/or health-enhancing properties. Such compounds include polyphenols, carotenoids, flavonoids, isoflavonoids, terpenoids, glucosinolates, phytoestrogens, and phytosterols. Studies on these phytochemicals have also shown positive pharmacological activities in human health [1].

**Figure 1:**
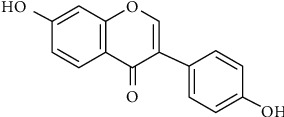
Chemical structure of daidzein.

Regarding phytochemical-rich plant sources, and to what concerns to isoflavonoid sources, soybeans and other leguminous plants are the main sources of active isoflavones genistein and daidzein [2]. Daidzein [7-hydroxy-3-(4-hydroxyphenyl)-4H-1-benzopyran-4-one] (Figure 1) is a naturally occurring phytoestrogen fitting into the category of nonsteroidal estrogens [3], with numerous pharmacological activities, such as antihemolytic, antioxidant, and anti-inflammatory activities [4, 5]. Daidzein can be found in soy-derived food products such as soy-based infant formulas, soy flour, textured soy protein, soy protein isolates, tofu, tempeh, and miso. In addition, soy flour is used for the fortification of other flours, including wheat, rice, and corn. The daidzein content of these products is quite variable, i.e., the daidzein amount is 22 mg in a half cup of miso, 15 mg in 3 ounces of tempeh, 8 mg in 3 ounces of tofu, and 7 mg in one cup of soy milk [6].

The chemical structure of daidzein is analogous to mammalian estrogens, making it a promising candidate for a dual purpose by substituting/hindering such hormones and their corresponding receptors. Hence, daidzein could be a therapeutic strategy for estrogen-dependent health conditions, such as breast [7] and prostate [8] cancer, diabetes, osteoporosis, and cardiovascular disease (CVD) [9]. However, daidzein also has other estrogen receptor- (ER-) independent biological activities, for instance, the ability to reduce oxidative damage, regulate the immune reaction [10], and induce apoptosis, directly linked to their anticancer effects [11]. Thus, such activities, along with minimum toxicity features, make daidzein a promissory compound for drug design. In this sense, the present review is aimed at providing an in-depth overview of daidzein's potential use to prevent or treat some burdening human health conditions. First, we focus on daidzein's pharmacodynamics and current limitations for its use. Then, we briefly describe some proposed mechanisms of action, and finally, we review its implications in human health showing the latest research in the field, namely, focusing on its ability to alleviate postmenopausal symptoms, and its potential anticancer and antiaging properties.

## 2. Daidzein Pharmacodynamics

**Figure 2:**
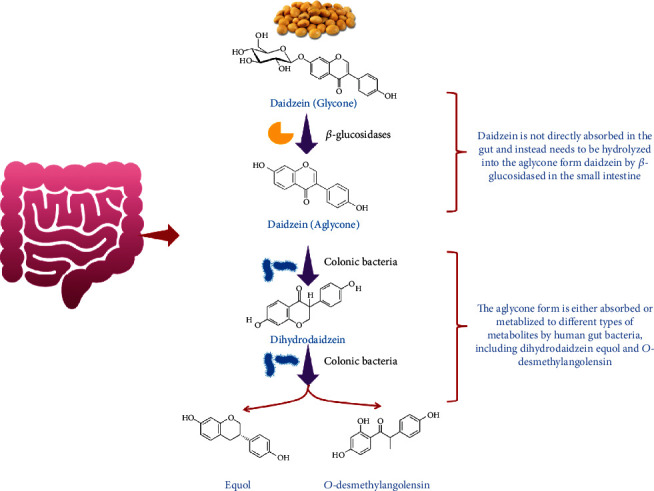
Biotransformation of daidzein in human gut.

Daidzein is predominantly found in soy and many unfermented foods not only in the form of daidzin, a glycoside conjugate [12, 13], but also as acetylglycoside and aglycone [14]. Daidzin is not directly absorbed in the gut and instead needs to be hydrolyzed into the aglycone form daidzein [15] by *β*-glucosidases in the small intestine [16]. The aglycone form is either absorbed or metabolized to different types of metabolites by human gut bacteria, including dihydrodaidzein [15], equol, and *O*-desmethylangolensin (*O*-DMA, a metabolite with no estrogenic activity) (Figure 2) [17]. This intestinal biotransformation is accomplished through several reactions, such as reduction, methylation and demethylation, hydroxylation, and C-ring cleavage [18]. The absorbed aglycone is metabolized mainly to glucuronidated derivatives and, to a lesser extent, into sulfated conjugates by phase I and II enzymes [19–21]. Then, these metabolites can be further metabolized in the liver or might be secreted into the bile and recycled [22]. Finally, both the unabsorbed daidzein and biliary derivatives that reach the colon undergo deconjugation by bacterial enzymes and are then reabsorbed or metabolized [18, 22–25].

Studies on daidzein absorption, bioavailability, distribution, and excretion are still limited [15, 26, 27], with data obtained so far revealing the appearance of a small peak in plasma around 1 h after ingestion, with daidzein being absorbed in the small intestine [28]. A larger peak appears after 5-8 h, from the conjugates recycling and colon absorption. Interestingly, daidzein can be found in plasma mostly in its conjugated form and a small proportion in the form of aglycone [29]. A clinical study showed that daidzein ingestion in the form of glucoside results in higher bioavailability than consuming the aglycone form [30], while a previous study showed contrary data [31]. These controversial results could be explained by differences in the type of glycosides or the influence of other isoflavones on their metabolism [32]. Regardless of these studies, it seems that daidzein reaches the maximum concentration in plasma approximately 7 h after ingestion [33], which appears to be directly linked to its complex absorption process. Finally, a study by Setchell et al. [33] suggested that almost all daidzein is rapidly absorbed and metabolized, as the excretion in feces and urine was minimal, although up to 30% of daidzein intake can be recovered in urine.

Regarding daidzein's biological activity, as well as of other isoflavones, it is highly dependent on their biotransformation, and huge differences have been stated in daidzein metabolism between humans, rats, and mice, which suggest that not all research studies regarding daidzein and its effects can be extrapolated to humans. In humans, glucuronides are the major plasma phase II metabolites and the proportion of plasma daidzein and other aglycones (0.5-1.3%) is significantly low compared to other animals [21].

Several factors, such as age, gender, or diet, have been described to influence the bioavailability of isoflavones in humans. For instance, the main source of isoflavones among the Asian population is fermented soy products, which contain isoflavones in the form of aglycones and can be directly absorbed. On the other hand, in the Western diet, the principal source is cooked soybeans, soy milk, and vegetable proteins, which contain the glucoside form [34]. Interestingly, an increased daidzein intake or its prolonged consumption appears not to change its bioavailability or pharmacokinetics (Setchell, Faughnan, Avades, Zimmer-Nechemias, Brown, Wolfe, Brashear, Desai, Oldfield, Botting and [3]).

Another important factor that determines daidzein bioavailability is the different food matrices used [26, 35]. Cassidy et al. [36] showed that daidzein absorption is faster when consuming soy milk, with glucoside conjugates, than solid soy food, with a significant difference of 2 h. Another study found that insoluble fiber, such as inulin, might increase the daidzein absorption [37, 38], partly due to bacterial growth stimulation [9]. However, there is a key aspect of daidzein metabolism that needs to be considered when studying its potential benefits. Plasma levels do not correlate well with the concentration that may effectively reach the different tissues. In fact, the quantification of isoflavones and their derivatives in human tissues is not usually determined and can vary in a large extent [24]. For instance, in humans, equol levels range between 22 and 36 nmol/kg in breast adipose tissue and 456-559 nmol/kg in glandular tissue [23, 39].

These complex daidzein pharmacokinetic features, along with its insolubility in water and oil, have blocked their use as a highly common compound in medicine or as a nutraceutical. Thus, several strategies have been developed to enhance daidzein's bioavailability, including emulsifying formulations or encapsulation with cyclodextrins [9]. For example, Peng et al. [40] designed fat-soluble derivatives by sulfonic acid esterification and stated that these are able to improve both daidzein cell uptake and its biological activities. Several techniques for modification of natural compounds are being developed and are discussed in other reviews [41, 42].

Equol (4′,7-isoflavandiol) is the daidzein metabolite that shows the strongest biological activity. Only a small percentage of the world population can metabolize daidzein to equol by gut bacteria [43]. The equol nonproducers, which have a prevalence between 80 and 90% in human subjects, convert a large part of daidzein into *O*-DMA [18]. Equol and *O*-DMA are likely produced by different bacterial taxa. Lu and Anderson [44] documented that only 30% of their study population presented equol conjugates in urine following soy administration, and no differences were reported regarding the type of diet. Furthermore, a prolonged soy intake led to the ability to produce equol in a small fraction of equol nonproducer women. In this regard, some known factors that limit the ability to produce equol are ethnicity and dietary habits [18]. For instance, up to 50-70% of the Asian population are equol producers, compared to only 20-30% of Western individuals [45]. Brown et al. [46] suggested that the ability to produce equol is developed during the first years of life, and it appears to be related to the diet composition in the early years, as they observed that breast-fed infants showed the lowest percentage of equol producers. Some other studies have tried to improve equol production by dietary habit modification. For example, Kruger et al. [47] analyzed the effects of a supplement of isoflavones with kiwifruit, expecting to see an improvement in equol production. Surprisingly, supplementation with kiwifruit had no effect on equol production and, in fact, attenuated the effects of isoflavone supplementation on reducing high-density lipoprotein (HDL) levels in postmenopausal women. Fructo-oligosaccharide supplementation also failed to increase equol production in postmenopausal Japanese women [48].

So far, most equol-producing bacterial strains belong to the family of *Coriobacteriaceae*, and they include *Adlercreutzia equolifaciens*, *Asaccharobacter celatus*, *Enterorhabdus mucosicola*, and *Slackia isoflavoniconvertens* and *Slackia equolifaciens*. Other equol-producing strains have also been identified, namely, *Bifidobacterium*, *Lactobacilllus*, *Lactococcus*, *Pediococcus*, and *Proteus* species [18]. The involvement of gut microbiota in daidzein metabolism highlights the importance of analyzing how diet and, specifically, how soy products can affect the balance of such microorganisms and understand the triggers for individual differences [43]. For instance, a recent study showed that isoflavone administration did not change the copy number of *Coriobacteriaceae* species in feces regardless of diet [18]. Iino et al. [49] reported that daidzein intake increased with age, as also the ability to produce equol. Interestingly, both equol producers and nonproducers held equol-producing bacteria, although the relative abundance of 2 species, namely, *A. celatus* and *S. isoflavoniconvertens*, was significantly higher in equol producers.

## 3. Daidzein Pharmacological Activities: Emphasis on Clinical Evidence

Epidemiological data suggest that isoflavone consumption may have health benefits and lower the risk of some age-related diseases, including osteoporosis, CVD, and several types of cancer, as well as reduce menopause-associated symptoms [18].  Table 1 resumes the different human studies reporting the effects of daidzein or isoflavones in several disorders.

In the Asian population, with a predominance of soy products in their diet, isoflavone intake can be up to 50 mg/day, while in Western countries, it is less than 2 mg, although it may be higher in menopausal women [109]. As a phytoestrogen, daidzein may induce its effects through the interaction with ERs, as it possesses a strong similarity with 17-*β*-estradiol (E2), the main female sex hormone. Two ER subtypes, namely, ER*α* and ER*β*, have been described with different tissue distribution and ligand-binding affinities. ER*α* is mainly found in breast and uterine tissues and has been associated with higher cell proliferation. On the other hand, ER*β* is the predominant isoform in the brain, bones, and blood vessels and is related to cell differentiation. Thus, to assess the overall effects of daidzein or any other phytoestrogen, the ER*α*/ER*β* ratio needs to be considered, as the cell response may considerably differ from one tissue to another [110, 111].

Both daidzein and equol are ER*α* and ER*β* agonists, with higher affinity for the latter, and can interfere with their signaling pathway. However, other ER-independent signaling mechanisms have been described, including protein kinase regulation, enzymatic inhibition, growth factor modulation, antioxidant activity, or epigenetic changes [111].

### 3.1. Daidzein and Allergies

Although estrogens are known to regulate the immune response, epidemiologic studies assessing the association between dietary isoflavones and allergic disorders are still limited. Miyake et al. [50] suggested that soy consumption and daidzein may reduce allergic rhinitis in Japanese women, although there was no dose-response effect. On the other hand, other products, such as tofu or fermented soybeans, showed no differences in allergic rhinitis prevalence. Nonetheless, it must be taken into account that soy is a strong food allergen, thus its consumption may be counterproductive when it comes to allergic disorders. Smith et al. [51] evaluated the soy isoflavone supplementation in poorly controlled asthmatic patients and found no differences in lung function between control patients and patients with isoflavone supplementation.

### 3.2. Effects of Daidzein on Osteoporosis and Menopausal Symptoms

Osteoporosis has a high incidence among menopausal women as estrogens regulate bone metabolism and ultimately prevent bone loss. Thus, the reduction in estrogens is associated with a higher risk of osteoporosis, and hormone replacement therapy has been proposed as a solution to reduce such risk [112]. In this regard, soy isoflavones have also been studied to prevent osteoporosis. Indeed, isoflavone supplementation for 4 and 6 months in postmenopausal women resulted in an increased bone density and improvement in bone resorption and formation biomarkers [52, 53]. Abdi et al. [57] reported in their systematic review that isoflavones may improve bone health and prevent mineral density loss in menopausal women.

Estrogens also exert direct effects on calcium homeostasis through ER-independent mechanisms. In fact, a correlation between estradiol and calcium levels has been described and inversely correlated with osteoporosis-associated fractures in humans [113]. Recently, Lu et al. [54] reported no changes in serum calcium levels with isoflavone pills uptake, containing 60 mg genistein and daidzein, 5 days/week for 2 years. However, a potential association was proposed between daidzein urinary excretion and serum calcium and chloride levels.

Pawlowski et al. [55] showed that a treatment with 105.23 mg total isoflavones/day, including genistein, daidzein, and glycitein, led to an increase in calcium retention in bones, although no differences were reported when equol producers and nonproducers were compared. On the other hand, Nayeem et al. [56] found a correlation between urinary isoflavone levels and decreased mineral density in women with low calcium levels.

Several studies have analyzed the effect of daidzein and equol in menopausal symptom reduction in women, like hot flashes and muscle and joint pain [2, 58]. Supplementation with 10 mg of equol 3 times/day reduced symptoms like anxiety, depression, and fatigue in postmenopausal women [59]. Other studies have also shown an improvement in some symptoms, including hot flash frequency, muscle stiffness, sweating, and renal function [58, 60, 63]. Interestingly, in several studies, equol-producing women showed a decrease in anxiety [59] and hot flash scores, as well as in sweating and fatigue [61], and hot flashes intensity [62] compared to equol nonproducing women. However, other studies have reported no benefits of daidzein or isoflavone supplementation in menopausal symptom reduction [64].

To address such controversy, some meta-analyses have been performed. Chen et al. [65] reported no evidence of improvement in the Kupperman Index, a questionnaire on menopausal symptoms, for women under a phytoestrogen treatment. However, based on the data obtained, the authors revealed that phytoestrogens seem to reduce hot flash frequency without having any marked side effects. Another meta-analysis reported such reduction in hot flashes with isoflavones, as well as other beneficial effects on vascular health, although they were not able to improve the urogenital symptoms [66]. Thus, taken together, such controversial results on the potential effects of daidzein and other isoflavones are presumably due to a lack of standardized protocol treatments, since different doses, study periods, supplement composition, and methods to determine the outcomes are used. Another proposed reason for this discrepancy in results is that most studies fail to distinguish equol producers from nonproducers and to determine the levels of free, unconjugated equol, which is presumably the main effector [114].

### 3.3. Daidzein and Cancer

Incidence and mortality rates of hormone-dependent tumors, such as breast, prostate, and ovarian cancer are considerably lower in Asia when compared to Western countries. This fact has been attributed to the higher soy isoflavone consumption in the Asian population, which has increased the interest in soy isoflavones for both prevention and treatment of such types of cancers [115]. However, some issues have yet to be resolved, such as the bioavailability of these compounds in the target tissue. Most studies show a dual effect of isoflavones on cancer depending on their concentration. Thus, tissue distribution and concentration must be determined to understand whether daidzein or other compounds may have beneficial or harmful effects in cancer [116]. For instance, Bolca et al. [23] analyzed the concentration of isoflavones in normal breast tissue following a dietary intervention increasing isoflavone intake, and they found that isoflavones may reach significant levels in breast to elicit a beneficial effect.

Several *in vitro* studies have described an anticancer effect for daidzein in different types of tumors [117–121]. Among the mechanisms described, daidzein was reported to induce apoptosis and cell cycle arrest in the SKOV3 ovarian cancer cell line [122] or induce epigenetic changes *in vivo* [123]. Furthermore, daidzein could modulate long noncoding RNA (lncRNA) expression in some cancer types, as several isoflavones have been reported to target these molecules [124].

The impact of soy on breast carcinogenesis has been widely evaluated. A meta-analysis conducted by Chi et al. [81] revealed that soy isoflavones may be associated with a lower incidence of breast cancer and that ER-negative breast cancer patients could benefit from an isoflavone supplementation. A decrease in breast cancer recurrence has been described for both soy consumption [67] and daidzein supplementation [68] in postmenopausal women. Interestingly, soy consumption has also been associated with a decreased expression of HER2/neu and PCNA in tumors, directly related to a more proliferative, malignant tumor phenotype [125]. On the other hand, Shike et al. [71] described a gene signature associated with higher cell proliferation in women with breast cancer with a supplement of soy protein, warning on the possible counterproductive effects of soy supplementation for breast cancer patients. Nevertheless, the American Association for Cancer Research recommends soy consumption in women, including those diagnosed with breast cancer [2]. In a meta-analysis, isoflavones showed a nonsignificative association with a decreased risk of breast cancer, as well as with either individual compounds as genistein, daidzein, and glycitein [69]. Soy isoflavone consumption has also been associated with a reduced risk of endometrial [77, 78] and ovarian cancer [79, 80]. However, other studies have found no effects of soy administration regarding endometrial health and cancer [72, 73]. In a recent review with meta-analysis, the authors suggested that phytoestrogens may play a role in breast cancer disease, although in other cancers, the evidence is too limited to draw this conclusion [70].

Prostate cancer incidence and mortality are significantly higher among North American and European men compared to Asian men. This difference has been attributed, in part, to the ability to produce equol, which is substantially higher among the Asian population [82]. Some studies have described a lower prostate cancer risk with soy isoflavone intake, although no changes in prostate-specific antigen (PSA) levels were observed under short-term treatments [2, 8, 74–76, 83, 126–128]. Zhang et al. [84] reported that while total isoflavones and equol were not correlated with prostate cancer risk, daidzein and other isoflavones could reduce the risk of developing this type of cancer.

Epidemiological data suggest that soy intake may have benefits for other types of cancer. For instance, phytoestrogen administration may be associated with a lower risk of colorectal cancer [85, 86]. Phytoestrogens have been described to increase the expression of ER*β* in normal colonic mucosa in humans [129], which could explain the protection against this type of cancer. However, since soy consumption is usually associated with healthier diet choices, this risk reduction may not be entirely due to daidzein and other soy components. Interestingly, Jiang et al. [34] detected that only in case-control and not in cohort studies the risk of colorectal cancer seemed to be reduced with isoflavone administration.

### 3.4. Daidzein and Cardiovascular Diseases

**Figure 3:**
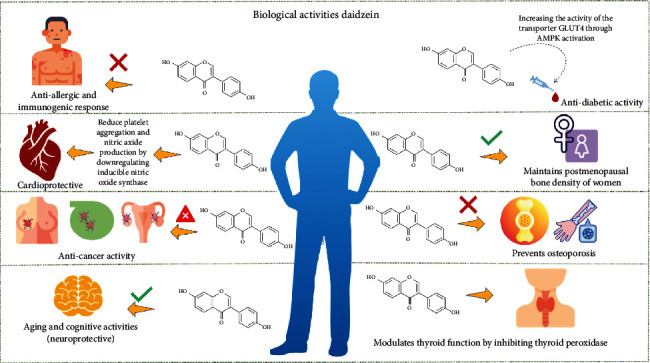
Various biological activities of the daidzein.

In animal models, daidzein was able to reduce platelet aggregation and nitric oxide production, suggesting a cardioprotective effect [130]. In this regard, daidzein has been reported to interfere with the inducible nitric oxide synthase (iNOS) expression pathway resulting in the downregulation of this enzyme (Figure 3) [131].

The first reports on the beneficial effects of soy products on human CV health were made more than two decades ago, with a meta-analysis showing that soy protein intake decreased total cholesterol (TC) and low-density lipoprotein- (LDL-) cholesterol levels [93]. Isoflavones have been found to enhance endothelial function and limit atherosclerosis progression [92], as well as lower blood pressure, improve lipid profile, and reduce oxidative stress and inflammation [132]. Daidzein administration only lowered serum triglycerides (TG) and uric acid, while the rest of the lipid profile and glucose remained unchanged. Interestingly, participants with a specific ER genotype were those who most benefit from this intervention [43]. Furthermore, equol has shown potential as an antiatherogenic agent and could prevent coronary heart disease [45].

Controversial results have been described in epidemiological studies analyzing the effects of isoflavones on coronary heart disease. The Shanghai Women's Health Study [87] and a Japanese cohort study [88] reported an inverse correlation between heart disease and dietary soy intake, while the Singapore Chinese Health Study [89] and the European Prospective study Into Cancer and Nutrition [90] showed no association. Zhang et al. [91] described a significant inverse correlation between coronary heart diseases and equol, but no effects were stated to soy isoflavones or their metabolites. On the other hand, another report suggested that the benefit for cardiovascular health is only seen in equol producers after 6 months of soy supplementation but not with the use of purified daidzein [63].

Finally, a meta-analysis performed by Glisic et al. [94] analyzed the effect of phytoestrogen on body weight and body composition in postmenopausal women. Phytoestrogen administration did not produce any changes in these parameters, although those participants with preexisting conditions such as diabetes or hyperlipidemia suffered an increase in body weight. Furthermore, daidzein could be associated with nonbeneficial effects in body composition. Miller et al. [133] suggested that gut microbiota could influence the incidence of obesity, as they reported that both peri- and postmenopausal women who did not produce *O*-DMA metabolite showed higher rates of overweight and obesity.

### 3.5. Effects of Daidzein on Aging and Cognitive Activities

Aging is usually associated with a decline in muscle mass and strength. Thomson et al. [95] analyzed the effects of soy intake on training resistance in older adults. Interestingly, they reported that those participants with soy protein supplementation did not gain as much muscle strength when compared to adults with regular protein or dairy protein intake. On the other hand, Orsatti et al. [96] reported a significant increase in muscle strength after 16 weeks of training resistance and soy supplementation in postmenopausal women.

Another hallmark of aging is a mild cognitive decline regarding learning, memory, and perception. The incidence of neurodegenerative diseases and dementia is also rapidly growing among the elderly population. Some studies have proposed estrogen therapy as a treatment for improving memory and preventing Alzheimer's disease in postmenopausal women [134]. Likewise, isoflavone administration may also improve cognitive functions and memory [97–100]. However, although a protective effect against Alzheimer's disease has been described in mice [135], following analysis of isoflavone supplementation effects in Alzheimer's disease patients, Gleason et al. [101] concluded that there were no significant benefits. Lately, Hernandez et al. [136] and Schneider et al. [102] tested PhytoSERM for 12 weeks in perimenopausal women, a mixture composed of genistein, daidzein, and equol. With a daily dose of 50 mg, the participants declared a reduction in menopausal symptoms and a better cognitive function, with no associated side effects. In this regard, more studies increasing the number of participants and analyzing the effects of PhytoSERM on cognitive decline are still undergoing.

### 3.6. Effects of Daidzein on Thyroid Function

Daidzein and other isoflavones are known enzymatic inhibitors and, theoretically, they may interfere with thyroid function as they inhibit thyroid peroxidase. However, several studies were measured thyroid function, and no special impact of isoflavones was found [103, 137]. Sosvorová et al. [104] confirmed that both genistein and daidzein are targets of thyroid peroxidase by detection of iodinated derivates of these isoflavones in human urine, although no effects were described in free thyroid hormones levels. Thus, there is no evidence that daidzein consumption could be harmful to thyroid gland disorders.

### 3.7. Daidzein and Diabetes

Isoflavones have also been studied for the treatment of diabetes. Interestingly, these compounds have the ability to modulate gut microbiota, which is altered in diabetes, and their potential use to prevent and manage this disease is currently being analyzed [138]. Some studies suggest that daidzein could enhance glucose and lipid metabolism, regulating glycemia and TC levels in animal models [139, 140] and increasing the activity of the transporter GLUT4 through AMPK activation [141]. Furthermore, the detection of equol in urine has been associated with a reduction in type 2 diabetes incidence among the Chinese population [105]. However, Gobert et al. [106] reported that isoflavones had no significant effect on glycemic control in patients with type 2 diabetes, and Ye et al. [107] found that daidzein improved neither insulin sensitivity nor glycemia following 6 months of treatment. Nevertheless, body weight control can be helpful for diabetes management. In this regard, isoflavones have shown potential to reduce fat accumulation and improve insulin resistance in animals [2, 142, 143]. Similarly, isoflavones could help in weight loss in humans [2, 108, 144], as these compounds have shown higher lipolytic potential [145]. Various biological activities of daidzein are shown in Figure 3.

### 3.8. Soy and Soy-Derived Metabolites in Children

Phytoestrogens may theoretically interfere with ER signaling in the developing brain of children or produce gut dysbiosis, although these results are controversial [146]. Soy-based formulas are often used for infants under certain circumstances, such as allergy and intolerance to milk, lactose intolerance, or galactosemia. Vandenplas et al. [147] evaluated the safety of these formulas and found that, although the levels of genistein and daidzein were higher in formula-fed infants, no harmful effects regarding anthropometric growth, immunity, cognition, or endocrine functions were found.

## 4. Conclusions and Future Perspectives

As mentioned before, daidzein has potent antioxidant and estrogenic activities, which has led to a wide interest in developing a functional food containing this compound. In adults, daidzein and other phytoestrogens are well-tolerated and have low levels of toxicity, while in infants, there are reports concerning their harmful effects. In the last years, there has been an increase in the consumption of soy products. For a better understanding of the properties of such soy products, it would be necessary to indicate, in addition to the quantity, the type of isoflavones that these products contain. Food processing technologies could affect both retention and distribution of different isoflavone isomers present in soy products. Both transformation and/or loss of some isoflavones, especially genistein and daidzein, may affect the nutraceutical characteristics of these soy products.

Although some of the benefits of isoflavones such as daidzein have been demonstrated, the side effects (for example the potential fertility problems among male humans) of long-lasting high consumption of these soy products need to be studied in a more in-depth way. In fact, clinical trial data are conflicting, showing both negative and positive effects of daidzein on human health. That is why a correct standardization and documentation of these clinical trials is essential to advance in the study of daidzein's beneficial effects on human health. Despite being possible to control all the independent variables in clinical trials, the ability of each individual to metabolize daidzein closely depends on individual's microbiota composition, the ability of this microbiota to assimilate the dose administered, and the different bioavailability of daidzein that could influence the data heterogeneity.

In the future, the use of genetic screening techniques could represent a great advance in personalized medicine. One of the uses of such techniques could be the assessment of the genetic predisposition of an individual to metabolize daidzein, which could initially help to select comparable groups for clinical trials and then filter the possible recipients of a treatment with daidzein, depending on individual's ability to metabolize this phytoestrogen. Moreover, the consumption of soy-rich products should be monitored by physicians, especially in cases of diseases for which daidzein is known to play an essential role, such as breast cancer [148].

## Figures and Tables

**Table 1:** Pharmacological activities of daidzein reported in human studies.

Pharmacological activity	Study type	Dose/type of treatment	Results	References
Antiallergic	Cross-sectional study	Regular soy products consumption	Possible reduction in allergic rhinitis incidence	[50]
	RCT	100 mg isoflavones/day, 24 weeks	No effects	[51]

Antiosteoporotic	CT	59.6 mg genistein + 15.6 mg daidzein/day, 6 months	Increase in bone mineral density in lumbar spine and decrease bone resorption biomarkers	[52]
	CT	70 mg isoflavones/day, 12 weeks	Increase in bone formation markers	[53]
	CT	60 mg genistein + 60 mg daidzein + 16.6 mg glycitein, 5 days/week, 2 years	No direct effects on serum calcium levels	[54]
	RCT	105.23 mg isoflavones/day, 50 days	Increase in calcium retention	[55]
	CT	136.6 mg isoflavones, 5 days/week, 2 years	Decrease in mineral bone density with low calcium levels	[56]
	SR	Different isoflavone extracts, 7 weeks up to 3 years	Bone health improvement and blocked mineral density loss	[57]

Menopausal symptoms reduction	RCT	10, 20, or 40 mg equol/day, 8 weeks	Reduced hot flash frequency and improved muscle and joint pain	[58]
	RCT	10 mg equol, 1 or 3 times/day, 12 weeks	Anxiety scores reduction	[59]
	CT	10 mg equol/day, 12 weeks	Reduced hot flash frequency and severity, improved sweating and muscle stiffness	[60]
	RCT	135 mg isoflavones/day, 1 week	Improved hot flashes, sweating and fatigue	[61]
	CT	100-200 mg isoflavones/day, 12 weeks	Improved hot flash intensity	[62]
	CT	63 mg daidzein/day, 6 months	Improved renal function	[63]
	RCT	150 mg isoflavones/day, 16 weeks	No effects on menopausal symptoms	[64]
	SR	Different isoflavones extracts, 3-12 months	Possible reduction in hot flash frequency	[65]
	SR	Different isoflavones extracts, 12 weeks to 4 years	Reduced hot flash frequency, improved vascular health, no effects on urogenital symptoms	[66]

Anticancer	Cohort study	Regular soy products consumption, 23 months	Reduced recurrence in tamoxifen-treated breast cancer patients	[67]
	Cohort study	Regular soy products consumption, 3.9 years	Reduced mortality and recurrence of breast cancer	[68]
	Meta-analysis	Regular isoflavones consumption	Decreased risk of breast cancer	[69]
	Review with meta-analysis	Regular phytoestrogen consumption	Possible role in survival from breast cancer	[70]
	CT	51.6 g soy protein/day (1.2 mg genistein/g protein, and 0.8 mg daidzein/g protein), 7-30 days	Increased markers of cell proliferation in breast cancer patients	[71]
	RCT	154 mg isoflavone soy protein/day, 3 years	No effects on endometrial cancer	[72]
	Cohort multicenter study	25 g isoflavone/day, 5 years	No effects on endometrial cancer	[73]
	RCT	80 mg isoflavones/day, 6 weeks	No changes in PSA in prostate cancer patients	[74]
	CT	47 mg isoflavones 3 times/day, 12 months	Reduced PSA levels in prostate cancer patients	[75]
	CT	60 mg isoflavones/day, 12 months	Reduced prostate cancer incidence in 65 years or older patients	[76]
	Meta-analysis	Regular soy products consumption	Reduced endometrial cancer risk	([77], [78])
	Meta-analysis and CT	Regular soy products consumption	Reduced ovarian cancer risk	([79], [80])
	Meta-analysis	Regular soy products consumption	Reduced mortality and recurrence of breast cancer	[81]
	Review	Regular soy products consumption	Reduced prostate cancer risk in equol producers	[82]
	Review	Regular soy products consumption	Reduced prostate cancer risk, no differences in PSA	[83]
	Review	Regular isoflavones consumption	Reduced prostate cancer risk for daidzein, not for equol	[84]
	Meta-analysis	Regular soy products consumption	Reduced colorectal cancer risk in women	[85]
	Review	Regular isoflavones consumption	No effect on stomach and colorectal cancer	[86]
	Review	Regular phytoestrogen consumption	Possible reduction in colorectal cancer risk	[86]

Cardioprotective	CT	Regular soy products consumption	Reduced CVD risk	[87]
	CT	Regular soy products consumption	Reduced myocardial infarction risk	[88]
	CT	Regular soy products consumption	No changes in CVD mortality	[89]
	CT	Regular phytoestrogen consumption	No changes in CVD risk	[90]
	CT	Regular isoflavones consumption	Reduced CVD risk	[91]
	CT	63 mg daidzein/day, 6 months	Improved renal function in postmenopausal women with prehypertension	[63]
	RCT	40-80 mg daidzein/day, 6 months	Reduced TG and uric acid	[43]
	Review	Regular soy products consumption	Reduced platelet aggregation and atherosclerosis	[92]
	Meta-analysis	Regular soy products consumption	Reduced TC, LDL-C and TG	[93]
	Review	Regular soy products consumption	Improved arterial stiffness and antiatherogenic effect	[45]
	Review	Different phytoestrogen supplementation	Improved body composition in postmenopausal women	[94]

Antiaging and cognitive promotion	RCT	27 g soy protein/day, 12 weeks	Decreased gain in muscle strength in older adults	[95]
	RCT	25 g soy/day, 16 weeks	Increase in muscle strength in postmenopausal women	[96]
	RCT	60 mg isoflavones/day, 6 months	Enhanced cognitive performance	[97]
	RCT	80 mg isoflavones/day, 4 months	Enhanced cognitive performance and memory	[98]
	CT	Regular phytoestrogen consumption	Improved verbal memory	[99]
	RCT	52 mg genistein + 36 mg daidzein + 3 mg glycitein daily, 2.5 years	Possibly improved visual memory	[100]
	RCT	100 mg isoflavones/day, 6 months	No beneficial effects for Alzheimer's disease patients	[101]
	CT	50 mg phytoSERM/day, 12 weeks	Possible enhancement of cognitive activity	[102]

Thyroid function	CT	80-120 mg isoflavones/day, 2 years	No changes in thyroid function	[103]
	CT	80 mg isoflavones/day, 3 months	No changes in thyroid function	[104]

Antidiabetic	CT	Regular isoflavones consumption	Decreased type 2 diabetes risk	[105]
	RCT	40 g soy protein/day, 57 days	No beneficial effects for glycemic control in diabetic patients	[106]
	RCT	50 mg daidzein/day, 12, 24 weeks	No beneficial effects for glycemic control or insulin sensitivity in diabetes patients	[107]
	RCT	10 mg equol/day, 12 weeks	Possibly improved glycemia control in overweight patients	[108]

CT: clinical trial; LDL: low density lipoprotein; RCT: randomized controlled trial; SR: systematic review; TC: total cholesterol; TG: triglycerides.

## Data Availability

The data used to support the findings of this study are available from the corresponding author upon request.
